# A preliminary exploration of the mechanism of endometrial cancer development induced by intestinal flora diversity and its metabolites

**DOI:** 10.3389/fcimb.2026.1815291

**Published:** 2026-08-12

**Authors:** Yuer Sun, Juan Li, Jiayu Chen, Haochen Peng, Yawen Shao, Zhenzhen Wu

**Affiliations:** 1 Department of Women’s Health, Gansu Provincial Maternity and Child Health Hospital (Gansu Central Hospital), Lanzhou, Gansu, China; 2 First School of Clinical Medicine, Gansu University of Chinese Medicine, Lanzhou, Gansu, China

**Keywords:** endometrial cancer, gut microbial ecosystem, intestinal flora, serum metabolites, tumorigenesis

## Abstract

**Background:**

Endometrial cancer (EC) is a highly prevalent gynecological malignancy increasingly affecting younger populations. While the gut microbiota significantly modulates tumor progression via organ-to-organ networks, the specific molecular mechanisms and microbial co-metabolites driving EC remain poorly understood. This study profiles gut microbiota alterations in EC patients to identify correlations between microbial lipid/bile acid metabolites and EC pathogenesis.

**Methods:**

A clinical cohort of 41 female participants (17 patients with histologically confirmed atypical endometrial hyperplasia or endometrioid adenocarcinoma, and 24 healthy controls) was prospectively enrolled. Fecal and serum samples underwent 16S rRNA gene sequencing (Illumina MiSeq) and LC-MS-based serum metabolomics, respectively. Spearman correlation analysis explored gut microbiota-metabolite associations.

**Results:**

The EC and control groups shared structural similarities, but the *Bacillota* / *Bacteroidota* (B/B) ratio was higher in the EC group (1.35) than the control group (1.19). LEfSe analysis identified *Megamonas, Megasphaera, Succinivibrio*, and *Veillonella* as the most influential genera in the EC group (LDA > 4.0). Metabolomics revealed significantly elevated levels of hypoxanthine, 2,4-dihydroxyacetophenone-5-sulfate, inosine, benzoylcholine, 20-hydroxyeicosatetraenoic acid (20-HETE), and theobromine in EC patients. KEGG enrichment analysis showed that glycerophospholipid and purine metabolism were the most significantly dysregulated pathways (*P* < 0.05 ), with fine-scale network dissection revealing pathological remodeling of glycerophospholipid metabolism. Spearman correlation between the top 5 differential genera and top 20 serum metabolites demonstrated functional cross-talk between specific gut microbiota and circulating lipid and porphyrin homeostasis.

**Conclusions:**

This study delineates distinct alterations in the gut microbiota and serum metabolomic profiles of EC patients, identifying *Megamonas* and *Klebsiella* as promising non-invasive predictive biomarkers. Targeting these specific taxa and their dysregulated downstream metabolites, such as protoporphyrin IX and 20-HETE, may offer novel avenues for early diagnosis and therapeutic intervention. Despite a modest sample size, these findings lay a foundation for future large-scale, mechanistic investigations into the gut-metabolism axis in EC.

## 1 Introduction

As one of the three primary malignancies affecting the female reproductive system, endometrial cancer (EC) imposes a substantial global health burden, with 2020 estimates indicating approximately 417, 000 new cases and 97, 000 deaths (Sung et al., 2021). Epidemiological data from the American Cancer Society (ACS) demonstrate that the annual incidence of EC rose by 1.2% from 2005 to 2014, accompanied by a distinct shift toward younger patient populations (Smith et al., 2018). Geographically, China accounts for a disproportionate share of this global burden—representing 23.7% of incident cases and 30.2% of deaths (Sung et al., 2021)—which underscores the disease’s status as a major public health crisis. Pathologically, atypical endometrial hyperplasia (AEH) serves as a critical premalignant precursor, with up to 40% of cases concurrently presenting with or progressing to well-differentiated endometrioid adenocarcinoma (Abu et al., 2023). Given that these conditions share overlapping etiological risk factors, identifying precise biomarkers for the early detection and risk stratification of these precursor lesions remains paramount to improving clinical outcomes.

Over the past decade, the cancer biology paradigm has expanded to acknowledge the host-microbiome continuum, wherein gut microbiota dysbiosis critically modulates systemic oncogenesis (Gupta S et al., 2019; Wang J et al., 2018). Within gynecological oncology, a compelling body of evidence links the gut microbiome to endometrial cancer (EC) via the “estrobolome”—the aggregate of enteric bacterial genes specialized in estrogen metabolism (Sepich-Poore GD et al., 2021; Cuevas-Ramos G et al., 2010). Extensive replication cohorts have established that microbial-derived β-glucuronidases deconjugate excreted estrogens within the intestinal lumen, driving their reabsorption into the systemic circulation and thereby enhancing long-term estrogen bioavailability (Yu et al., 2026; Mattavelli E et al., 2026). This persistent hyperestrogenic state fuels the uncontrolled proliferation of endometrial epithelial cells, anchoring the classic microbial-estrogen-carcinogenesis axis. Beyond this endocrine pathway, emerging evidence suggests that the gut microbiota exerts direct, multi-omic regulatory control over the endometrial microenvironment. Recent framework integrations indicate that microbial-derived bioactive metabolites—specifically short-chain fatty acids (SCFAs) and secondary bile acids—transcend the gastrointestinal tract to induce localized epigenetic modifications and immune reprogramming within the endometrium (Wang J et al., 2018). Consequently, the scientific consensus has shifted from viewing the gut microbiota merely as a passive estrogen regulator to recognizing it as an active orchestrator of host metabolic and inflammatory homeostasis.

Paradoxically, translating these mechanistic insights into non-invasive diagnostic modalities remains heavily impeded by discordant data and geographical variations among existing cohorts. Although certain multi-ethnic studies highlight discrete taxonomic signatures associated with endometrial lesions (Taira T et al., 2015; Bernstein et al., 2011), the directional shifts of pivotal candidate biomarkers—particularly *Bacteroides* and *Lactobacillus* species—exhibit pronounced inconsistency across diverse patient populations (Baker JM et al., 2017). These discordant findings stem largely from unadjusted inter-individual confounders, such as localized dietary habits, body mass index (BMI), and ethnicity, which exacerbate microbial heterogeneity and obfuscate the definitive etiological trajectory of endometrial carcinogenesis. Consequently, a validated, universally applicable microbial-metabolic signature capable of robustly differentiating EC from healthy controls has yet to be established.

To address these pivotal knowledge gaps, the present study couples 16S rRNA gene sequencing with untargeted serum metabolomics to comprehensively delineate the gut microbiota–host metabolic interactome across a clinical continuum spanning healthy controls and endometrial cancer (EC) patients. By decoding the compositional and functional dynamics synchronized with endometrial lesion progression, we aimed to resolve the precise taxonomic perturbations and metabolic dysregulation landscapes underlying oncogenesis. Ultimately, this rigorous multi-omics integration seeks to reconcile existing cross-cohort inconsistencies, thereby offering robust, translational biomarkers for the early detection and risk stratification of endometrial cancer.

## 2 Materials and methods

### 2.1 Research subjects

Following institutional review board (IRB) approval and the acquisition of written informed consent, a clinical cohort was prospectively recruited at Gansu Maternal and Child Health Hospital (Gansu Provincial Central Hospital) between February and August 2023. The disease cohort comprised patients diagnosed with pathologically confirmed atypical endometrial hyperplasia (AEH) or endometrioid adenocarcinoma in the Department of Gynecology. The control group consisted of healthy female volunteers undergoing routine health screenings during the same period. To ensure unbiased selection among the 82 eligible candidates, a computer-assisted stratified random sampling approach was executed utilizing the SPSS random number generator, stratified by clinical grouping (EC vs. healthy controls). The finalized study cohort comprised 41 female participants, consisting of 17 patients in the EC group and 24 in the healthy control group. Eligibility and Exclusion Criteria. To be eligible for inclusion in the disease group, patients were required to meet the following criteria: (1) age 18–75 years; (2) diagnosis of AEH according to the WHO classification, or pathologically confirmed endometrioid adenocarcinoma staged in accordance with the 2009 FIGO criteria post-standard hysterectomy. Healthy controls were required to have matching demographic profiles and no history of uterine or gynecological pathology. Candidates were excluded if they presented with any of the following confounding conditions: (1) a history of other concurrent malignancies or hormone-related systemic disorders; (2) current pregnancy or lactation; (3) prior history of oncological interventions, including chemotherapy, radiotherapy, or immunotherapy; (4) recent administration (within 3 months) of antibiotics, probiotics, immunosuppressants, or hormonal therapies; (5) active or recent gastrointestinal disorders (e.g., chronic diarrhea) within 3 months; or (6) specialized or restrictive dietary habits, such as strict vegetarianism.

### 2.2 Specimen collection

Prior to surgery or during routine health examinations, fresh fecal specimens (≈ 1.5–3.0 g) were aseptically collected from patients and healthy volunteers using sterile instruments. These specimens were immediately transferred into 7.0-mL sterile centrifuge tubes and rapidly cryopreserved at -80°C. Concurrently, peripheral blood samples (2.5 mL) were obtained via venipuncture and delivered to the clinical laboratory for processing. To isolate serum, the whole blood was centrifuged at 4, 000 rpm (approximately 2, 000×g) for 5 min at room temperature. The resulting serum supernatant was subsequently aliquoted into duplicate labeled cryovials, cataloged with unique identification barcodes, and stored at -80°C pending downstream multi-omics analyses.

### 2.3 Bacterial colony detection and difference analysis with linear discriminant analysis

Library Preparation and High-Throughput Sequencing. Genomic DNA samples (30 ng) of verified quality were mixed with matching fusion primers to optimize PCR amplification parameters. Following amplification, the PCR products were purified using Agencourt AMPure XP magnetic beads and eluted in elution buffer to complete library construction. The fragment size distribution and concentration of the resulting libraries were assessed using an Agilent 2100 Bioanalyzer. Validated libraries that passed quality control based on their insert sizes were subsequently subjected to high-throughput sequencing. Sequence Quality Control and Filtration. Raw sequencing reads were filtered to remove low-quality sequences using a sliding-window approach. Specifically, a 25-bp window was established; if the average quality score within the window dropped below 20, the downstream bases were truncated from that position. Sequences were completely discarded if the truncated read length was less than 75% of the original length. Furthermore, reads contaminated with adapters (defined as a default adapter sequence overlapping by 15 bp with the reads, allowing up to 3 mismatches), reads containing ambiguous bases (“N”), and low-complexity sequences (defined as ≥ 10 consecutive identical bases) were excluded. Samples were demultiplexed based on unique primers and barcodes, permitting zero mismatches between barcode sequences and sequencing reads. Bioinformatics and Statistical Analysis. Taxa exhibiting significant shifts in relative abundance during treatment were identified using the DESeq2 package (version 1.12.4). Significantly differentially abundant taxa were screened based on False Discovery Rate (FDR) correction, with thresholds set at |log_2_fold-change| > 2.0 and *P* < 0.05. Subsequently, linear discriminant analysis effect size (LEfSe) software was employed to estimate the effect size of each microbial component on the differential variance, where a log-linear discriminant analysis (LDA) score > 3.0 was defined as statistically significant.

### 2.4 Serum metabolite pretreatment and metabolite identification

Metabolite Extraction and Mass Spectrometry Detection. Extracted metabolites were analyzed using a high-resolution mass spectrometry system operated in both positive and negative electrospray ionization modes. Peak extraction, based on the precursor mass-to-charge ratio (*m/z*) and retention time of the metabolites, was executed using XCMS software to identify specific metabolic features, with primary chromatographic peak areas integrated for relative quantification. Metabolite Identification and Annotation Confidence. Initial metabolite identification was performed using the open-source metaX software by matching experimental *m/z* values against the Human Metabolome Database (HMDB) and Kyoto Encyclopedia of Genes and Genomes (KEGG) databases. To address the inherent challenge of isobaric compounds and structural isomers within these repositories—which frequently result in a single *m/z* value corresponding to multiple candidate molecules—tentative annotations were rigorously validated. This structural confirmation was achieved by cross-referencing the secondary mass spectrometry (MS/MS) fragmentation patterns of the sample metabolites with reference MS/MS spectra from authentic chemical standards, thereby ensuring a high level of annotation confidence. Data Preprocessing and Normalization. Individual feature intensities across all biological and quality control (QC) samples were extracted via XCMS. Statistical quality control was conducted using metaX, whereby low-quality peaks (defined as ion features missing in > 50% of QC samples or > 80% of actual biological samples) were systematically filtered and discarded. The remaining missing values were subsequently imputed. Finally, the dataset was normalized using Probabilistic Quotient Normalization (PQN) to yield the final, authenticated metabolite identification and relative quantification matrices.

### 2.5 Statistical analysis of metabolomics data

Metabolomic data preprocessing and downstream statistical analyses were executed through a structured, multi-step bioinformatics workflow. During initial quality control (QC), metabolic features with missing values exceeding 50% in QC samples or 80% in experimental samples were systematically excluded. The remaining missing values were then imputed using the K-Nearest Neighbors (KNN) method. To control for potential sample dilution effects, data normalization was performed via Probabilistic Quotient Normalization (PQN), utilizing the median peak area ratio of the QC samples as the reference calibration. To identify robust differential metabolites, multivariate Partial Least Squares-Discriminant Analysis (PLS-DA) was coupled with univariate statistical modeling. Specifically, metabolites were defined as significantly altered if they concurrently satisfied the following strict criteria: a Variable Importance in the Projection (VIP) value > 1.0 derived from the PLS-DA model, a two-tailed *P*-value < 0.05 calculated by the Wilcoxon rank-sum test (W-test), and a fold-change (FC) > 1.5 or < 0.67. Biological functional annotation and pathway integration were subsequently conducted using the Kyoto Encyclopedia of Genes and Genomes (KEGG) database (https://www.genome.jp/kegg/pathway.html). To determine whether the identified differential metabolites were significantly enriched within specific biological cascades, a hypergeometric test was deployed, with a significance threshold established at a Benjamini-Hochberg adjusted *P*-value < 0.05.

### 2.6 Statistical methods

Clinical and baseline characteristics were analyzed using SPSS software (version 26.0). The normality of continuous variables was assessed via the Shapiro-Wilk test. Normally distributed variables were expressed as mean ± standard deviation (SD) and compared across groups using one-way analysis of variance (ANOVA) followed by the Tukey-Kramer *post-hoc* test. Non-normally distributed variables were presented as median and interquartile range (IQR) and analyzed using the Kruskal-Wallis test. Categorical variables were expressed as frequencies (percentages) and compared using Pearson’ s chi-square test.For high-dimensional, non-normal, and compositional microbiome data, rigorous bioinformatics pipelines were deployed. Differential taxonomic abundance was determined using Linear Discriminant Analysis Effect Size (LEfSe) and the DESeq2 package. To stringently control for type I errors arising from multiple hypothesis testing, raw *P* -values generated by LEfSe, DESeq2, and downstream Spearman rank correlation analyses (evaluating associations between specific gut microbiota and serum metabolites) were corrected using the Benjamini-Hochberg false discovery rate (FDR) method. Furthermore, a five-fold cross-validation approach was applied to evaluate the robustness of the Spearman correlation coefficients by iteratively partitioning the dataset and calculating the mean and SD of the coefficients across all validation folds. For general clinical parameters, a two-tailed *P* < 0.05 was considered statistically significant. For omics datasets and correlation analyses, a multiple-testing adjusted *P* -value (FDR *Q* -value) < 0.05 was mandated to denote statistical significance.

## 3 Results

### 3.1 Baseline characteristics of the study population

The body mass index (BMI) of patients in the EC group was significantly higher than that of the control group, with seven patients (41.2%) presenting a BMI exceeding 28 kg/m^2^, thereby meeting the criteria for obesity. A significant difference in menopausal status was observed between the two groups (*P* < 0.01), with 10 patients (58.8%) in the EC group being postmenopausal (**Table 1**). According to the 2018 International Federation of Gynecology and Obstetrics (FIGO) staging system, 15 of the 17 EC patients were classified as stage I, and 1 was stage III; the remaining patient was diagnosed via an external pathological consultation and lacked formal surgical staging. Regarding histological grading, 13 cases (76.4%) exhibited well-differentiated tumors (G1), and 4 cases (23.5%) showed moderately differentiated tumors (G2).

**Table 1:** Clinicopathological characteristics of participants.

Characteristics	EC group (n = 17)	N group (n = 24)	P-value
Age(years)^a^	50.52 ± 7.26	33.87 ± 7.28	0.805
Height(m)^a^	1.59 ± 0.04	1.61 ± 0.04	0.160
Weight(kg)^a^	71.41 ± 16.09	55.13 ± 6.53	<0.001
BMI(kg/m2)^a^	27.88 ± 5.55	21.31 ± 2.81	<0.001
Waist(cm)^a^	95.32 ± 24.38	74.27 ± 6.51	0.012
Hips(cm)^a^	96.05 ± 18.67	92.72 ± 5.87	0.005
Diabetes^b^	4(23.5)	-	<0.001
Fasting Blood Sugar(mmol/L)^a^	5.48 ± 1.28	5.01 ± 0.43	0.001
High Blood Pressure^b^	8(47.0)	-	<0.001
Systolic blood pressure(mmHg)^a^	134.58 ± 22.45	108.62 ± 11.95	0.016
Diastolic blood pressure(mmHg)^a^	86.17 ± 12.17	66.87 ± 8.08	0.088
Menopause^b^	10(58.8)	-	<0.001
Pregnancy^a^	2.29 ± 1.31	1.25 ± 0.94	0.403
Number of births^a^	1.70 ± 0.84	0.95 ± 0.80	0.404
2018 FIGO stage^b^			
I	15(88.2)	-	
II	0	-	
III	1(5.8)	-	
IV	0	-	
Tumor grade^b^			
G1	13(76.4)	-	
G2	4(23.5)	-	
G3	0	-	

^a^Measures are expressed as Mean ± SD.

^b^Categorical variables are expressed as numerical values (%).

### 3.2 Tags splicing and OTU clustering analysis

**Figure 1:**
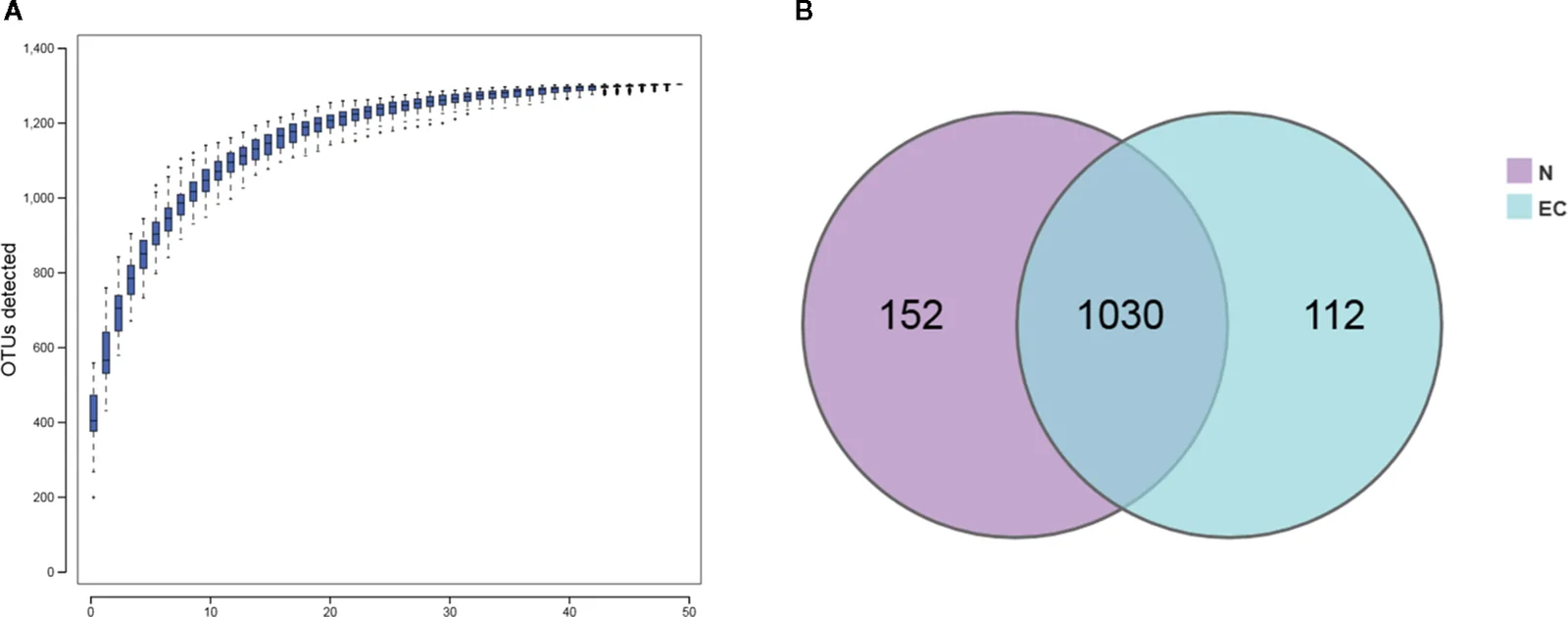
**(A)** Species accumulation curve. The horizontal axis is the number of samples sequenced and the vertical axis is the OUTs detected. As shown in the figure, the sample size designed in this study is large enough that the sequencing results accurately reflect the species information of most microorganisms in the current samples. **(B)** Venn diagram. Venn diagram of operational taxonomic units (OTUs) showed the number of common and unique OTUs, and the similarity and overlap of OTUs between the two groups. N, controlgroup; EC, endometrial cancer group.

Sequence Assembly and OTU Clustering. Paired-end reads generated from sequencing were merged using FLASH software (v1.2.11) based on their overlap relationships. High-quality tags within the target hypervariable region were extracted under stringent splicing parameters: a minimum overlap length of 15 bp and a maximum allowable mismatch rate of 0.1 within the overlap region. Operational taxonomic units (OTUs) were generated by clustering the processed tags at a 97% sequence similarity threshold using UPARSE. Following the removal of chimeric sequences via UCHIME (v4.2.40), all remaining high-quality tags were mapped back to the representative OTU sequences using the ‘usearch global’ method to construct the final OTU abundance profiles for each sample. Sequencing Depth and Core OTU Distribution. As illustrated by the species accumulation curve (**Figure 1A**), the core species richness approached a clear plateau as the sequencing depth expanded, demonstrating that both the sequencing depth and sample size in this study were sufficient to capture the vast majority of microbial diversity within the cohorts. A comparison of the two clinical groups via a Venn diagram (**Figure 1B**) revealed a substantial overlap, with 1, 030 shared OTUs accounting for 79.6% of the total microbial abundance. Additionally, the control (N) group exhibited a higher number of unique OTUs (n = 152) compared to the EC group (n = 112).

### 3.3 Analysis of the composition of the intestinal flora in the EC group and the control group

**Figure 2:**
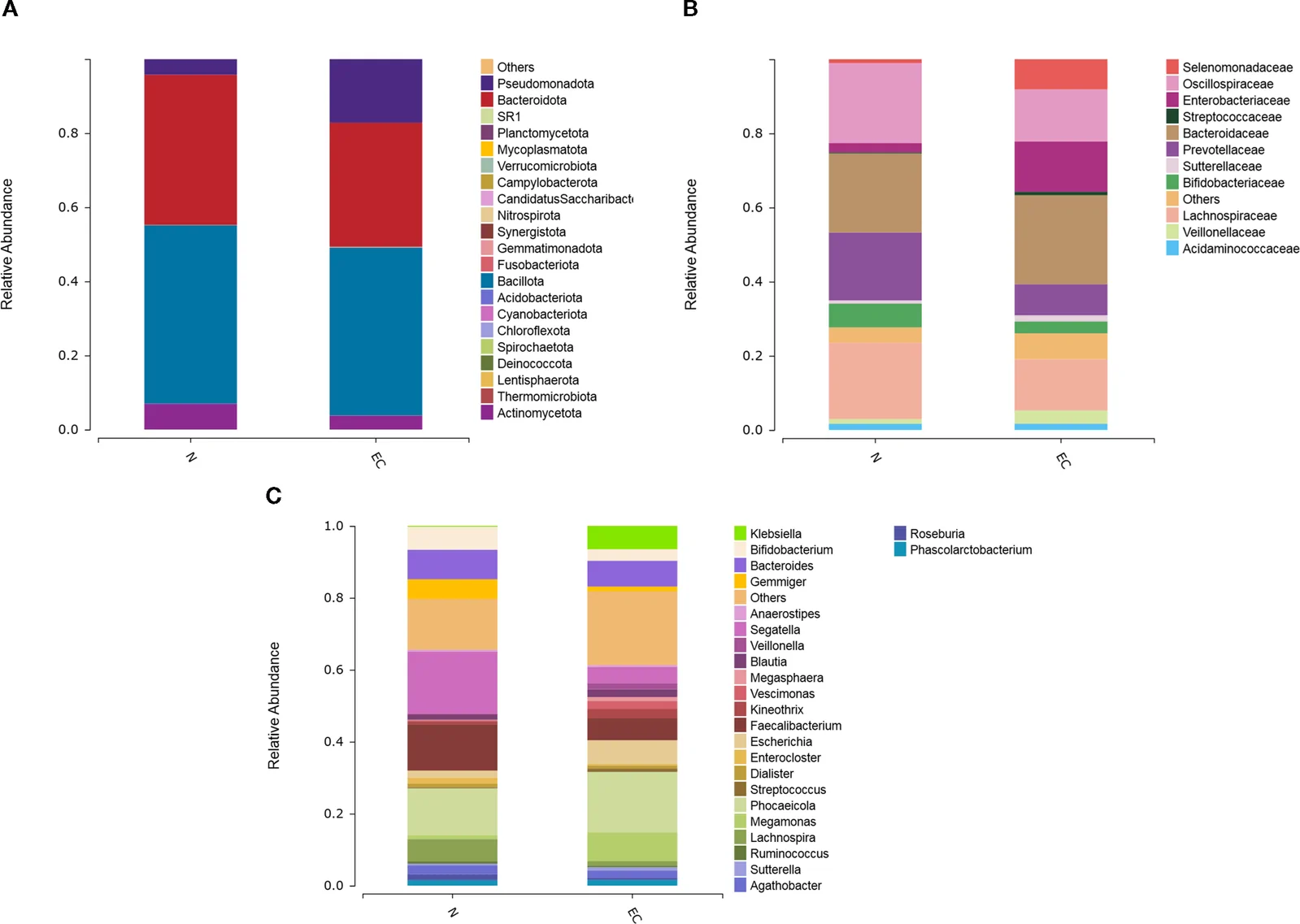
**(A)** Bar plot of gut microbiota composition at the phylum level. Unannotated taxa at this classification level and species with relative abundances below 0.5% in the samples were grouped into”Others”. **(B)** The analysis of species differences at the family level. **(C)** The analysis of species differences at the genus level.

At the phylum level (**Figure 2A**), *Bacillota* and *Bacteroidota* were the core dominant phyla in the gut microbiota of the N and EC groups. The relative abundances of *Bacillota* in the N and EC groups were 48.14% and 45.24%, respectively, while the relative abundances of *Bacteroidota* were 40.55% and 33.48%, respectively. However, the *Bacillota*/*Bacteroidota* ratio was skewed in the N and EC groups: the *Bacillota/Bacteroidota* ratio in the EC group (*B/B* = 1.35) was higher than in the N group (*B/B* = 1.19). A higher *B/B* ratio is associated with inflammation or abnormalities in metabolic pathways such as tryptophan and lipid metabolism, and may play a role in the progression of EC. Additionally, *Pseudomonadota* abundance was higher in the EC (17.1%) groups than in the N group (4.2%), but the difference between the groups was not statistically significant (*P >*0.05).

At the family level (**Figure 2B**), *Bacteroidaceae* was the dominant family in all three groups (N group: 21.32%, EC group: 23.99%). The abundance of *Enterobacteriaceae* was elevated 5.4-fold in the EC group (13.59%) compared to the N group (2.52%). *Selenomonadaceae* was significantly enriched in the EC group (H = 8.12, *P* = 0.003), *Selenomonadaceae* is associated with inflammatory cytokines, such as increased levels of IL-1β and TNF-α (Boulangé CL et al., 2016) suggesting that these bacteria play corresponding regulatory roles at different stages of endometrial lesions.

At the genus level (**Figure 2C**), although *Phocaeicola* was the most dominant genus in both groups (N group: 13.72% ± 5.21%, EC group: 17.27% ± 8.43%), no significant differences in its abundance were observed between the groups (*P* = 0.343).The EC group showed significant enrichment of *Megamonas* (H = 7.48, *P* < 0.001) and *Succinivibrio* (H = 0.65, *P* = 0.003). The relative abundance of *Escherichia* in the EC group (5.91% ± 2.15%) was higher than in the N group (1.94% ± 0.67%). The abundance of *Megamonas* in the EC group (7.48% ± 3.21%) was 7.0-fold higher than in the N group (0.94% ± 0.35%). The EC group exhibited a distinctive enrichment of *Megamonas*. Further LDA analysis is needed to identify gut microbial biomarkers associated with the progression of endometrial lesions.

### 3.4 Alpha diversity analysis of intestinal flora in EC and control groups

**Figure 3:**
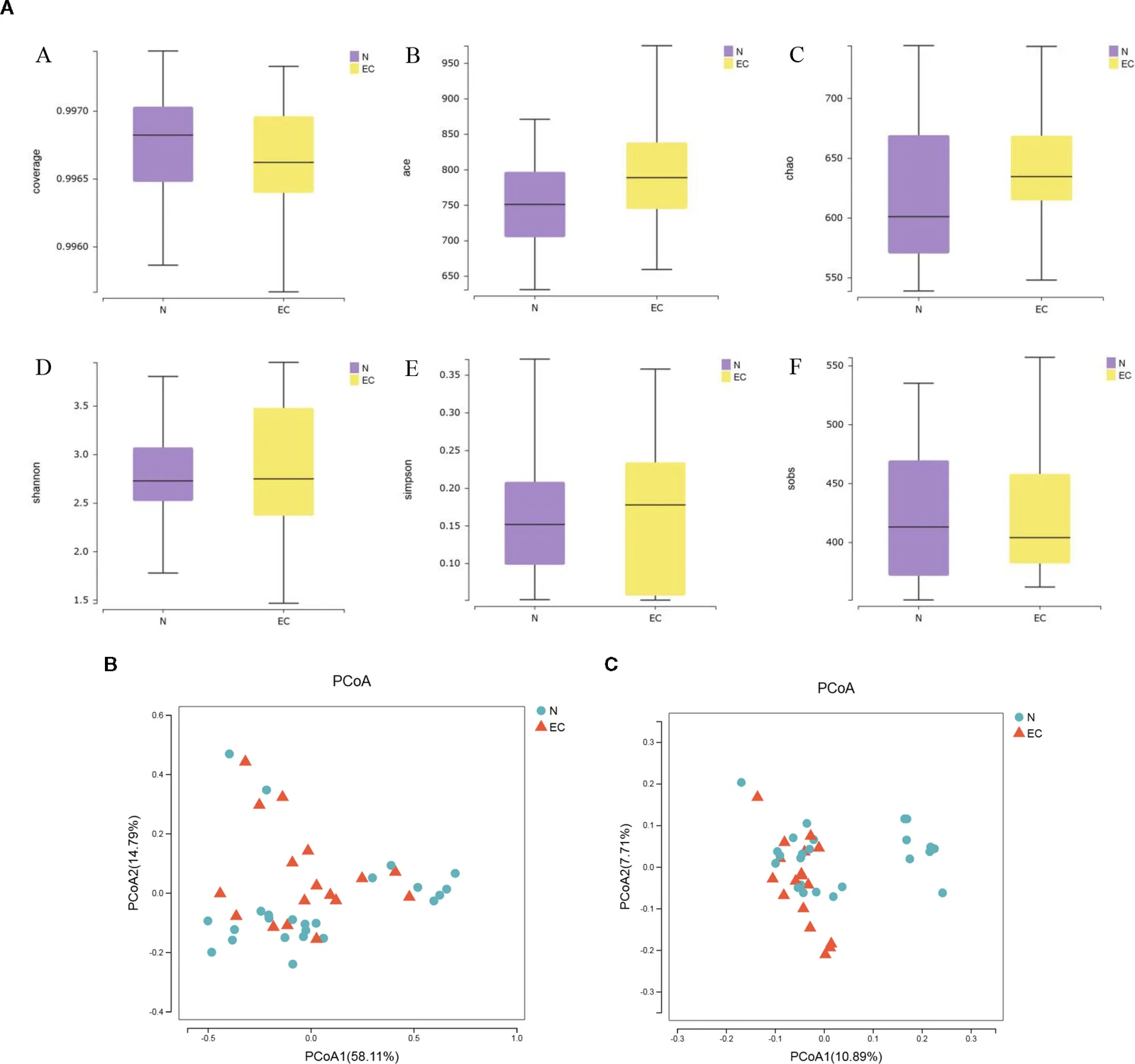
**(A)** Box plot of intergroup differences in α-diversity. **(A)** Coverage index, **(B)** Ace index, **(C)** Chao index, **(D)** Shannon index, **(E)** Simpson index and **(F)** Sobs. **(B)** Principal coordinates analysis (PCoA) of gut microbiota based on weighted Bray-Curtis distance. The x- and y-axis scales represent the projection coordinates of the sample points on the two-dimensional plane. **(C)** Principal coordinates analysis (PCoA) of gut microbiota based on unweighted Bray-Curtis distance.

Alpha diversity metrics, including the observed species (Sobs), Chao, ACE, Shannon, and Simpson indices, were calculated to evaluate microbial richness and evenness. Differences in alpha diversity across the study groups were visualized via box plots generated using R software, and statistical significance was determined using the non-parametric Kruskal-Wallis rank-sum test, with a two-tailed *P* < 0.05 considered statistically significant. As illustrated in **Figure 3A**, the Good’ s coverage index approached 99.7% for both cohorts, demonstrating that the sequencing depth was sufficient to capture nearly the entire gut microbial community. Regarding community richness, the EC group exhibited a slight upward trend in baseline bacterial abundance compared to the control group; however, this variance did not reach statistical significance, as indicated by the ACE (*P* = 0.233) and Chao (*P* = 0.086) indices. Similarly, community diversity and evenness remained statistically comparable between the two cohorts, with no significant differences observed in either the Shannon or Simpson indices (*P* = 0.317).

### 3.5 Beta diversity analysis of intestinal flora in EC group and control group

Beta diversity was assessed via QIIME2, utilizing both unweighted and weighted UniFrac distances to quantify phylogenetic dissimilarities between samples. Principal Coordinate Analysis (PCoA) based on the unweighted UniFrac distance revealed a clear and significant separation between the N and EC groups (*P* < 0.001; **Figure 3B**). In contrast, PCoA utilizing the weighted UniFrac distance demonstrated no significant clustering between the two cohorts (*P* = 0.227; **Figure 3C**). This discrepancy indicates that while the overall microbial profiles and dominant taxa remained structurally similar, the shifts differentiating the EC and N groups were primarily driven by low-abundance or rare opportunistic taxa.

### 3.6 Analysis of intestinal flora in EC group and control group

**Figure 4:**
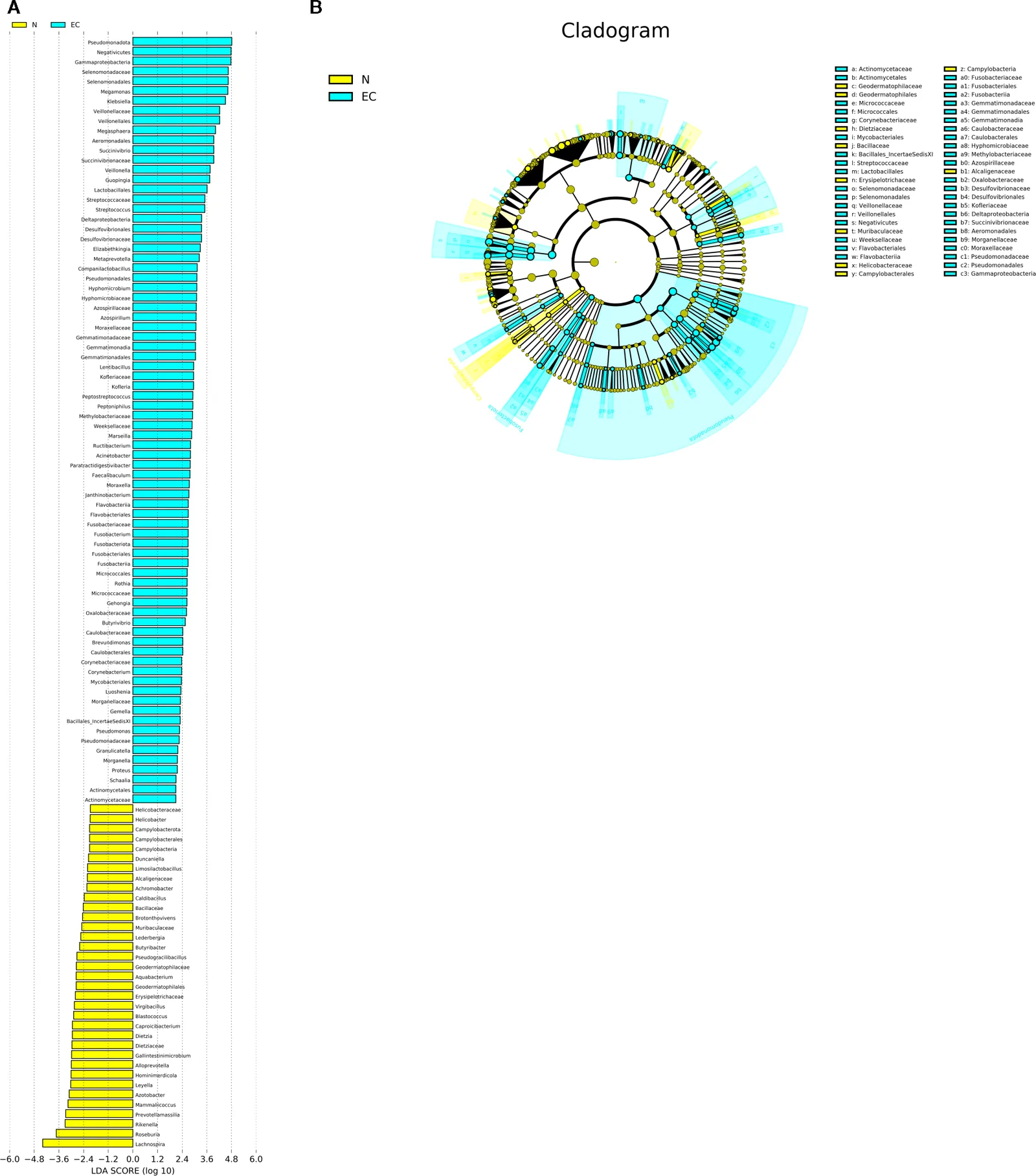
**(A)** LEfSe LDA bar plot. The plot displays significantly different taxa (biomarkers) with LDA scores greater than the predefined threshold, which is set to 3.0 by default. The length of each bar represents the LDA score. **(B)** LEfSe cladogram. Nodes in different colors represent microbial taxa that play an important role in the corresponding group. Each colored node indicates a biomarker, and the legend in the upper right corner shows the names of these biomarkers. Yellow nodes indicate taxa that are not significantly associated with any group. From the center to the outermost ring, the circles represent taxa at the phylum, class, order, family, and genus levels, respectively.

To identify specific bacterial taxa characterizing this microbial shift, a linear discriminant analysis effect size (LEfSe) analysis was performed, complemented by taxonomic cladogram mapping. As illustrated in **Figure 4A**, Megamonas*, Megasphaera, Succinivibrio*, and *Veillonella* exerted the most substantial influence on the gut microbiota structure of the EC group, all exhibiting an LDA score > 4.0. The hierarchical distribution of these differentially abundant taxa across multiple taxonomic levels (from phylum to genus) is visualized in a cladogram (**Figure 4B**), where colored nodes highlight the key biomarker lineages that significantly differentiate the two study cohorts.

### 3.7 Analysis of serum metabolites and metabolic enrichment

**Figure 5:**
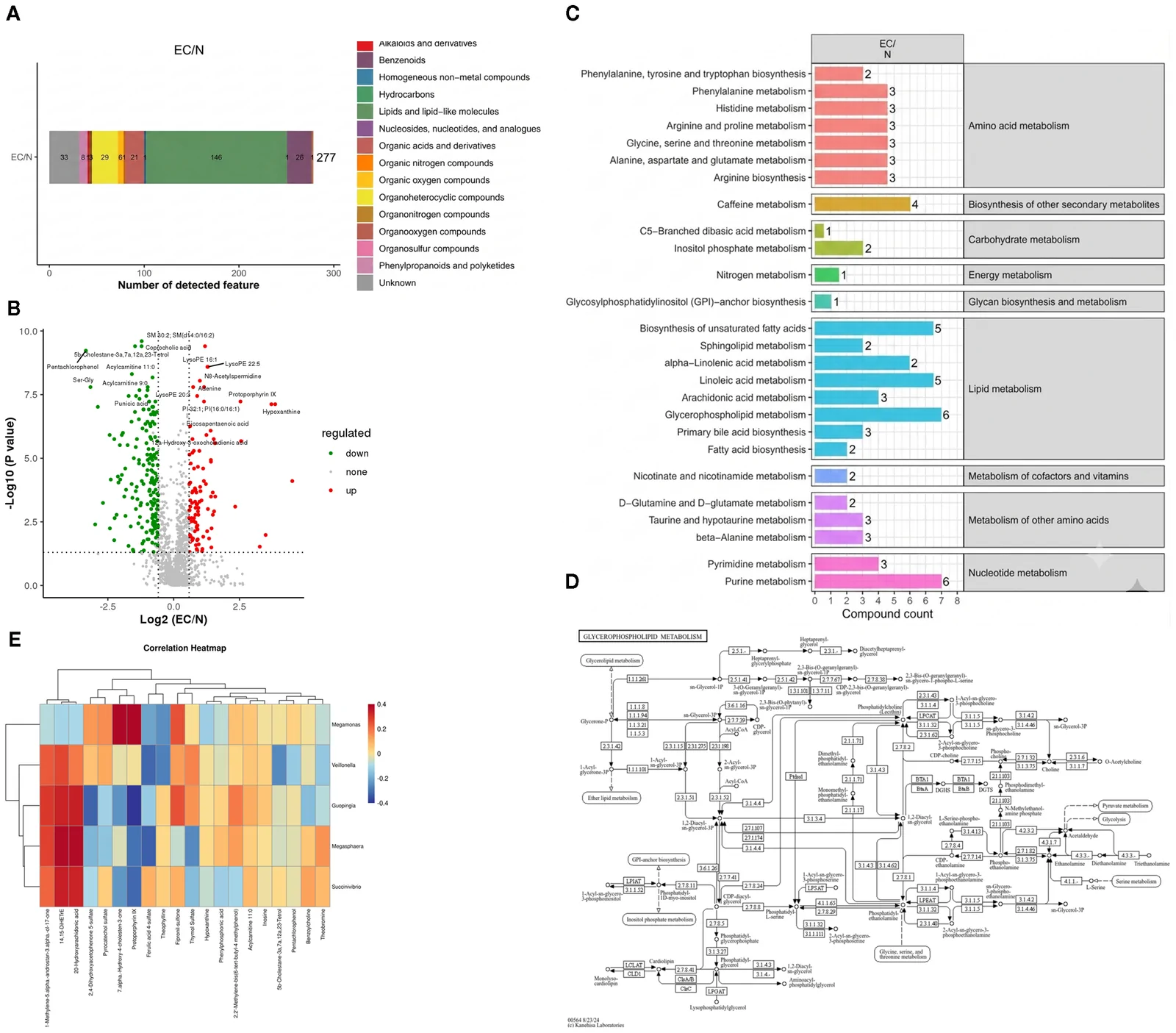
**(A)** Number of differential metabolites between the EC group and the N group. The x-axis represents the number of differential metabolites, and the metabolite categories are based on secondary identification. **(B)** Volcano plot of secondary differential metabolites between the EC group and the N group. The x-axis represents the log^2^ fold change of average intensities between the two phenotypes, while the y-axis shows the statistical significance (–log^10^ P value). A higher y-axis value indicates greater statistical significance. **(C)** KEGG enrichment pathway map of secondary differential metabolites between the EC group and the N group. The x-axis indicates the number of differentially expressed genes (DEGs) involved in each pathway, where bar length is proportional to the gene count. The y-axis details the specific pathways within the metabolism hierarchy. **(D)** Glycerophospholipid metabolism pathway. This pathway illustrates the key steps in glycerophospholipid metabolism, including the synthesis and interconversion of phosphatidylcholine, phosphatidylethanolamine, and phosphatidylserine. The pathway is sourced from the KEGG database (https://www.kegg.jp/). **(E)** Spearman correlation heatmap between differential bacteria and metabolites in endometrial cancer group. The x-axis represents significantly different serum metabolites, and the y-axis represents differential bacterial genera identified by LEfSe. The color gradient indicates the Spearman correlation coefficient (r).

To elucidate the circulating metabolic landscape of endometrial cancer, we characterized differential metabolites and their enriched pathways within the EC group. Based on stringent selection criteria (VIP > 1, *P* < 0.05, and fold-change [FC] > 2.0 or < 0.5), hierarchical clustering was performed to visualize the significantly altered metabolic profiles. A total of 4, 390 differential metabolic features were identified between the two cohorts, comprising 1, 493 upregulated and 2, 897 downregulated features. Quality control (QC) assessments confirmed high data reproducibility. Classification analysis revealed that the predominant differential metabolites originated from lipids and lipid-like molecules (**Figure 5A**). Further stratification by VIP scores indicated that the abundance of hypoxanthine, 2, 4-dihydroxyacetophenone 5-sulfate, inosine, benzoylcholine, 20-hydroxyarachidonic acid, and theobromine was markedly elevated in the EC group (**Figure 5B**). Subsequently, KEGG enrichment analysis demonstrated that glycerophospholipid metabolism and purine metabolism were the most profoundly dysregulated pathways in EC patients (*P* < 0.05;**Figure 5C**).

Granular dissection of the glycerophospholipid metabolism network further illustrated its pathological rewiring in EC (**Figure 5D**). Within this cascade, glycerol-3-phosphate is initially catalyzed by lysophosphatidic acid acyltransferase (LPAAT) to generate 1-acylglycerol-3-phosphate, which sequentially serves as a precursor for phosphatidylcholine (PC) and phosphatidylethanolamine (PE). The homeostatic synthesis of these core structural lipids is tightly orchestrated by cytidine diphosphate-choline (CDP-choline) and lysophosphatidylcholine acyltransferase (LPCAT). Concurrently, phosphatidylserine (PS) is synthesized via lysophosphatidylserine acyltransferase (LPSAT) and undergoes reciprocal interconversion with other lipid fractions through the alternative CDP-lipid pathway. Crucially, this hyperactivated metabolic axis “governs host” cell membrane biosynthesis, oncogenic signal transduction, and cellular energy expenditure, thereby fueling cancer cell proliferation, local invasion, and systemic immune evasion to accelerate the initiation and progression of EC.

### 3.8 Correlation study between intestinal flora and serum metabolites

To explore the functional intersections between the gut microbiota and host metabolism, Spearman correlation analysis was performed between the top 5 differentially abundant microbial genera (selected by LDA scores) and the top 20 serum metabolites (selected by VIP values). As shown in **Figure 5E**, *Megamonas* exhibited moderate correlations with protoporphyrin IX, 20-hydroxyarachidonic acid (20-HETE), 14, 15-dihydroxyeicosatrienoic acid (14, 15-DiHETrE), and 2, 4-dihydroxyacetophenone 5-sulfate (with *r* values ranging from -0.433 to 0.340). Additionally, *Megasphaera* correlated moderately with 14, 15-DiHETrE, 20-HETE, and ferulic acid-4-sulfate (*r* range: -0.328 to 0.379), while *Succinivibrio* displayed positive associations with 7α-hydroxy-4-cholesten-3-one and protoporphyrin IX (*r* range: 0.387 to 0.389). Furthermore, *Veillonella* showed a distinctive correlation with ferulic acid-4-sulfate (*r* = -0.316).

Among the key metabolic components within this interactive network, three functional classes stood out: (a) 20-HETE and (b) 14, 15-DiHETrE, which are lipid mediators generated via the cytochrome P450 epoxygenase pathway that actively modulate vascular tone and systemic inflammation; and (c) protoporphyrin IX (an organic heterocyclic compound per the HMDB database), which serves as the immediate precursor in the heme biosynthetic pathway. Collectively, these results suggest that specific EC-associated enteric taxa engage in functional cross-talk with circulating lipid and porphyrin homeostasis, although the exact regulatory mechanisms require further experimental validation.

## 4 Discussion

### 4.1 The landscape of gut dysbiosis in EC and paradigm-shifting novelty

Although a critical knowledge gap remains regarding the role of the intestinal microbiota in endometrial cancer (EC)—largely owing to geographical constraints and limited sample sizes in current pilot cohorts (Chen et al., 2025)—accumulating evidence underscores a profound systemic metabolic-immunological linkage between mucosal ecosystems and gynecological malignancies. Moving beyond static, descriptive inventories of microbial taxonomy, our study establishes a comprehensive multi-omics network that integrates taxonomic fluctuations with circulating serum metabolomic footprints (Zhao et al., 2022).

The central novelty of this investigation lies in translating the “gut-endometrium axis” from a speculative physiological concept into a quantifiable, mechanically evaluable framework. While previous studies have largely focused on macro-level microecological shifts or generalized dysbiosis (Herrera et al., 2024), we achieve high-resolution mapping that directly links specific opportunistic taxa to pro-inflammatory, immunosuppressive, and pro-angiogenic circulating lipid metabolites. This multidimensional analysis provides a granular molecular explanation of how intestinal dysbiosis exerts long-range, systemic oncogenic effects, thereby laying a robust framework for future microecology-based interception strategies in EC.

### 4.2 Disruption of host homeostasis and the pathophysiological paradigm of the B/B ratio

Under optimal physiological conditions, the complex human gut microbiota is predominantly governed by the phyla *Bacillota* (formerly *Firmicutes*) and *Bacteroidota*, which engage in structural and immunological cross-talk to preserve host epithelial tight junction integrity and maintain mucosal immune privilege (Joos et al., 2025; Abdeen et al., 2025). In our clinical cohort, the significant elevation of the *Bacillota/Bacteroidota* (*B/B*) ratio in EC patients serves as a distinct indicator of homeostatic barrier disruption. Mechanistically, an altered *B/B* ratio represents a metabolic hallmark of compromised mucosal permeability and chronic structural degradation of the intestinal epithelium, frequently manifested as “leaky gut” (Holeček M, 2020). The immediate functional consequence of this compromised barrier is the uncontrolled, paracellular translocation of potent pathogen-associated molecular patterns (PAMPs)—most notably Gram-negative bacterial lipopolysaccharides (LPS)—from the intestinal lumen directly into the portal and systemic circulation. This persistent leakage establishes chronic, low-grade systemic endotoxemia, serving as a critical upstream instigator of systemic inflammation. Rather than remaining localized, this pro-inflammatory milieu continuously delivers cytokines (such as IL-6 and TNF-α) and Toll-like receptor (TLR) agonists to distant mucosal tissues. Specifically, this distal signaling primes the endometrium, perpetuating a microenvironmental imbalance and sustained hyperproliferation that ultimately promotes genomic instability, thereby driving neoplastic transformation.

### 4.3 Mechanistic links intertwining key taxa and angiogenic/inflammatory metabolites

Our fine-scale taxonomic and LEfSe cladogram dissections identified highly specific, dominant genera within the Bacillota and Pseudomonadota phyla that actively drive EC pathogenesis through discrete metabolic circuits. This selectively disrupts the conventional, oversimplified narrative that labels the entire Bacillota phylum as strictly benign, fiber-fermenting commensals (Xing et al., 2024).

#### 4.3.1 The megamonas and megasphaera co-oncogenic axis

The enrichment of *Megamonas* and *Megasphaera* observed within the EC cohort correlated robustly with circulating metabolites involved in dysregulated lipid and systemic bile acid pathways—specifically 7α-hydroxy-4-cholesten-3-one, protoporphyrin IX, 14, 15-DiHETrE, and 20-hydroxyarachidonic acid (20-HETE). Biologically, 20-HETE and 14, 15-DiHETrE function as potent lipid mediators. Upon entering the vascular compartment, these eicosanoids stimulate endothelial cell migration, survival, and capillary tube formation, potentially through the aberrant activation of endothelial VEGFR or MAPK/ERK signaling cascades, thereby promoting tumor-associated neoangiogenesis to sustain expanding neoplastic tissues. Concurrently, dysregulated bile acid intermediates, such as 7α-hydroxy-4-cholesten-3-one, possess cytotoxic properties that aggravate mucosal inflammation via chronic activation of the NF-κB signaling pathway. Synergistically, these microbial taxa and their metabolic byproducts shape an immunosuppressive, pro-inflammatory microenvironment that shields the developing endometrial tumor from host immunosurveillance, ultimately driving clonal progression.

#### 4.3.2 Opportunistic dynamics and estrogen-driven reprogramming of Succinivibrio

Similarly, the pronounced overrepresentation of *Succinivibrio* and its taxonomic family *Selenomonadaceae* underscores the opportunistic, context-dependent nature of the EC-associated gut microbiota. Although typically existing as non-pathogenic commensals under effective host immunosurveillance, members of the *Selenomonadaceae* family undergo pathogenic expansion when host immunity is progressively subverted by a developing malignancy. Intriguingly, our finding regarding the marked enrichment of *Succinivibrio* diverges from previous multi-cancer paradigms, which typically characterize this taxon as a fiber-fermenting commensal that reinforces the mucosal barrier (Lu et al., 2021). We hypothesize that this functional divergence is primarily driven by the hyper-estrogenic milieu defining the physiological baseline of EC patients. High circulating estrogen levels alter intestinal substrate availability, mucosal immune signaling, and luminal pH, while modulating the estrobolome—the aggregate of enteric bacterial genes capable of metabolizing estrogens. This altered microenvironment likely induces a “functional reprogramming” event within *Succinivibrio*, triggering a transition from fiber fermentation to a pro-inflammatory phenotype that exacerbates barrier degradation, facilitates translocation, and fuels the systemic inflammatory cascade driving EC progression (Wallace et al., 2010).

### 4.4 Limitations and future directions

Despite the novel mechanistic insights provided by this investigation, several limitations warrant acknowledgment. First, the cross-sectional design and relatively modest sample size of our clinical cohort preclude the establishment of definitive causal or chronological relationships between gut dysbiosis and endometrial carcinogenesis (Yang et al., 2025). Consequently, it remains challenging to ascertain whether the identified microbial signatures act as primary etiological drivers or represent secondary consequences of the host tumor burden. Second, although individual gut microbiota profiles are inherently heterogeneous, potential confounding variables—including dietary habits, body mass index (BMI), menopausal status, geographical location, lifestyle, and medication exposures—were not fully controlled for. To address these limitations and move from association to causality, future research should prioritize large-scale, prospective, and longitudinal multi-center cohorts. Integrating stratified sampling or mixed-effects models within these larger cohorts will be essential to rigorously account for inter-individual confounding factors across diverse populations. Furthermore, mechanistic and interventional studies using germ-free (GF) mouse models subjected to humanized fecal microbiota transplantation (FMT) are imperative to validate the underlying biological pathways. Ultimately, establishing these causal links will facilitate the development of innovative, microbiota-targeted therapeutics, personalized interventions, and risk-stratified preventive strategies for endometrial cancer.

## 5 Conclusions

Taken together, this study delineates distinct alterations in the gut microbiota structure and serum metabolomic profiles of EC patients, identifying *Megamonas* and *Klebsiella* as promising non-invasive predictive biomarkers for endometrial cancer. Targeting these specific microbial taxa and their dysregulated downstream metabolites, such as protoporphyrin IX and 20-HETE, may offer novel avenues for early diagnosis and therapeutic intervention in EC. Although constrained by a modest sample size, our findings lay a conceptual foundation for future large-scale cohorts and mechanistic investigations aimed at elucidating the precise causal pathways and molecular networks through which the gut-metabolite axis modulates EC pathogenesis.

## Data Availability

The datasets for this study can be found in the Genome Sequence Archive at the National Genomics Data Center (Nucleic Acids Res 2024), China National Center for Bioinformation/Beijing Institute of Genomics, Chinese Academy of Sciences (GSA: PRJCA034591) [https://ngdc.cncb.ac.cn/gsa].
